# Deletion of AA9 Lytic Polysaccharide Monooxygenases Impacts A. nidulans Secretome and Growth on Lignocellulose

**DOI:** 10.1128/spectrum.02125-21

**Published:** 2022-06-06

**Authors:** César Rafael Fanchini Terrasan, Marcelo Ventura Rubio, Jaqueline Aline Gerhardt, João Paulo Franco Cairo, Fabiano Jares Contesini, Mariane Paludetti Zubieta, Fernanda Lopes de Figueiredo, Fernanda Lima Valadares, Thamy Lívia Ribeiro Corrêa, Mario Tyago Murakami, Telma Teixeira Franco, Gideon J. Davies, Paul H. Walton, Andre Damasio

**Affiliations:** a Department of Biochemistry and Tissue Biology, Institute of Biology, University of Campinas (UNICAMP), Campinas, São Paulo, Brazil; b Brazilian Biorenewables National Laboratory (LNBR), Brazilian Center for Research in Energy and Materials (CNPEM), Campinas, São Paulo, Brazil; c Interdisciplinary Center of Energy Planning, University of Campinas (UNICAMP), Campinas, São Paulo, Brazil; d Department of Chemistry, University of York, York, United Kingdom; Tufts University

**Keywords:** LPMO, AA9, oxidative enzymes, fungal biology, carbohydrate metabolism, sugarcane lignocellulose, CAZymes

## Abstract

Lytic polysaccharide monooxygenases (LPMOs) are oxidative enzymes found in viruses, archaea, and bacteria as well as eukaryotes, such as fungi, algae and insects, actively contributing to the degradation of different polysaccharides. In Aspergillus nidulans, LPMOs from family AA9 (*An*LPMO9s), along with an AA3 cellobiose dehydrogenase (*An*CDH1), are cosecreted upon growth on crystalline cellulose and lignocellulosic substrates, indicating their role in the degradation of plant cell wall components. Functional analysis revealed that three target LPMO9s (*An*LPMO9C, *An*LPMO9F and *An*LPMO9G) correspond to cellulose-active enzymes with distinct regioselectivity and activity on cellulose with different proportions of crystalline and amorphous regions. *An*LPMO9s deletion and overexpression studies corroborate functional data. The abundantly secreted *An*LPMO9F is a major component of the extracellular cellulolytic system, while *An*LPMO9G was less abundant and constantly secreted, and acts preferentially on crystalline regions of cellulose, uniquely displaying activity on highly crystalline algae cellulose. Single or double deletion of *An*LPMO9s resulted in about 25% reduction in fungal growth on sugarcane straw but not on Avicel, demonstrating the contribution of LPMO9s for the saprophytic fungal lifestyle relies on the degradation of complex lignocellulosic substrates. Although the deletion of *An*CDH1 slightly reduced the cellulolytic activity, it did not affect fungal growth indicating the existence of alternative electron donors to LPMOs. Additionally, double or triple knockouts of these enzymes had no accumulative deleterious effect on the cellulolytic activity nor on fungal growth, regardless of the deleted gene. Overexpression of *An*LPMO9s in a cellulose-induced secretome background confirmed the importance and applicability of *An*LPMO9G to improve lignocellulose saccharification.

**IMPORTANCE** Fungal lytic polysaccharide monooxygenases (LPMOs) are copper-dependent enzymes that boost plant biomass degradation in combination with glycoside hydrolases. Secretion of LPMO9s arsenal by Aspergillus nidulans is influenced by the substrate and time of induction. These findings along with the biochemical characterization of novel fungal LPMO9s have implications on our understanding of their concerted action, allowing rational engineering of fungal strains for biotechnological applications such as plant biomass degradation. Additionally, the role of oxidative players in fungal growth on plant biomass was evaluated by deletion and overexpression experiments using a model fungal system.

## INTRODUCTION

A complex set of carbohydrate-active enzymes (CAZymes) devoted to the plant biomass degradation are produced by saprotrophic fungi (1) including many Aspergillus species, and this set includes a diversity of redox-active auxiliary activity enzymes (AAs) such as lytic polysaccharide monooxygenases (LPMOs) and cellobiose dehydrogenases (CDHs) (2–10).

LPMOs are copper-dependent enzymes that utilize an oxidative mechanism to cleave the glycosidic bonds of polysaccharides in the presence of an external electron donor and O_2_ (11). LPMOs can also utilize H_2_O_2_ as the oxidizing cosubstrate (12, 13). LPMOs from family AA9 (LPMO9s) are numerous in some fungal genomes (14), with members exhibiting activity on cellulose, glucans, and xylans (15, 16). Electrons to reduce LPMOs can be provided by various sources such as small chemical compounds (e.g., ascorbate and monophenols) or enzymes, including the AA3_1 CDHs (17). Fungal CDHs have been shown to provide electrons for the redox-mediated oxidative cleavage of cellulose (18, 19), while also being involved in lignin degradation (20, 21).

Many studies have reported the functional characterization of LPMOs (22–27) and CDHs (28–30) and their functional partnership has been demonstrated *in vitro* (31–35). The importance of fungal CDHs in biomass degradation has been investigated in *Trametes versicolor* (36), Neurospora crassa (33, 37), and Podospora anserina (38). Different aspects of LPMOs’ biological role have been investigated in fungi (39–43), however, to the best of our knowledge, the implication of these enzymes for fungal growth on lignocellulose has not been demonstrated.

Aspergillus nidulans is a model organism for genetic and experimental studies (44, 45) harboring nine LPMO9s and two CDH encoding genes in its genome (46). The diversity of putative LPMO9s in this fungus (*An*LPMO9s) along with the CDH (*An*CDH1) has been verified by different approaches (2, 5, 25), and partially characterized (25, 47). However, it has not been characterized *in vivo*.

In this work, the time-resolved expression of CAZymes by A. nidulans was assessed in response to crystalline cellulose and lignocellulosic substrates derived from sugarcane crops. Among several CAZymes, six LPMO9s were secreted overtime along with a cosecreted CDH. Based on the secretion profile, three candidates were obtained by homologous expression in A. nidulans and biochemically characterized. The biological role of these enzymes in the model organism A. nidulans was investigated by constructing mutants carrying single, double, and triple deletions for LPMO9s and CDH, and by overexpression of LPMO9s.

## RESULTS

### The secretion of LPMO9s and other CAZymes are time and substrate dependent.

**FIG 1:**
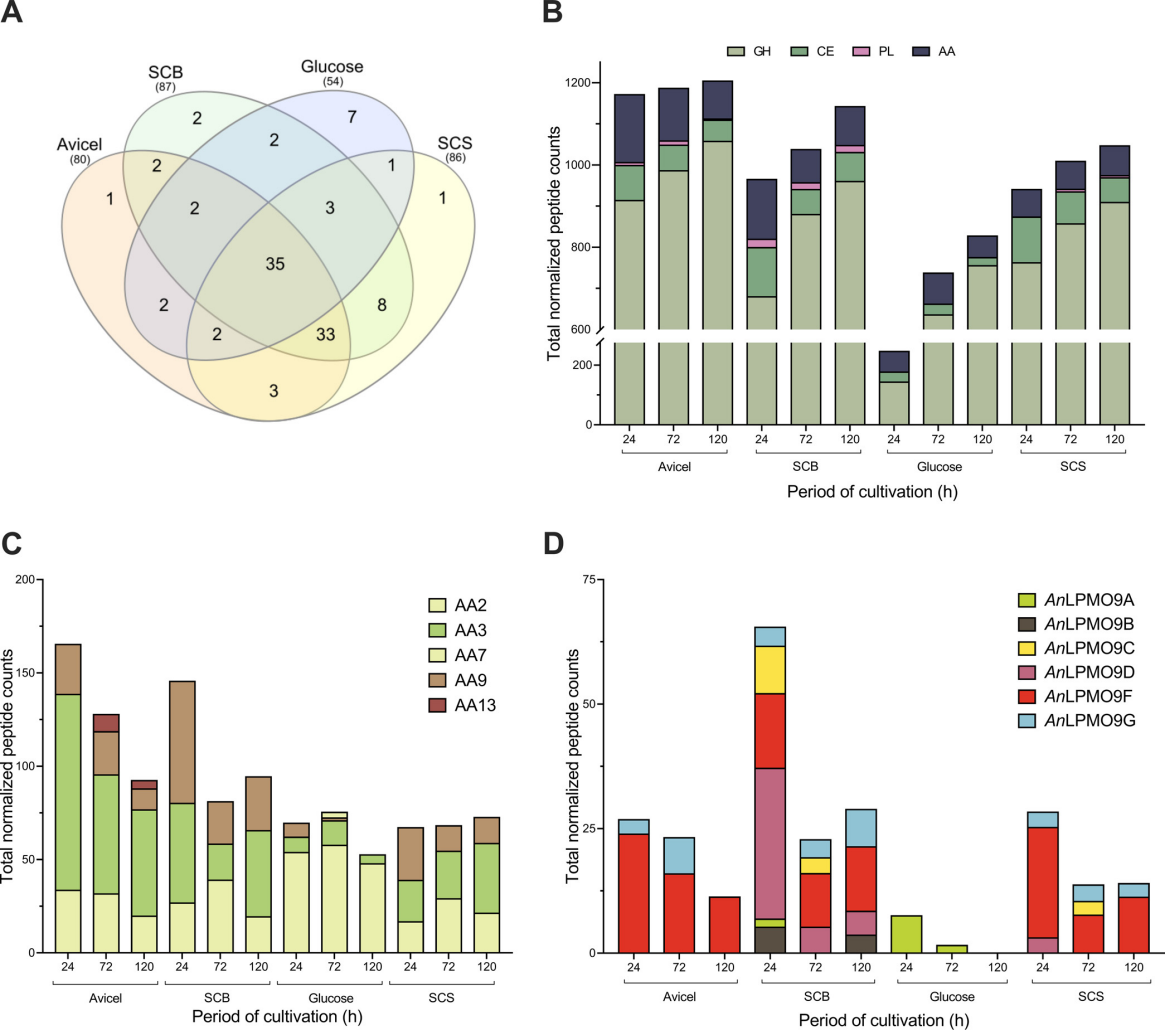
Analysis of proteins identified by MS in the extracellular proteomes of A. nidulans A773 cultivated on different substrates. (A) Venn diagram depicting the number of unique and common proteins identified in the secretomes produced on different substrates; (B), (C), and (D) quantitative analysis of CAZymes identified on each substrate overtime - abundance was estimated by the sum of peptide counts from normalized data. Secretion of (B) CAZymes, (C) Auxiliary Activities, and (D) AA9 LPMOs. SCB - sugarcane bagasse, SCS - sugarcane straw.

To understand better the A. nidulans response to lignocellulose in the environment, secretomes (set of extracellular proteins) induced on crystalline cellulose (Avicel) or agroindustry-derived lignocellulose, such as steam-exploded sugarcane bagasse (SCB) and sugarcane straw (SCS), were investigated by mass spectrometry (MS). Out of the 253 proteins identified in all secretomes, 104 proteins presented CAZymes domains, corresponding to 64 glycoside hydrolases (GHs), 3 pectin lyases (PLs), 15 carbohydrate esterases (CEs), and 22 AAs (Table S1). Most of CAZymes were commonly secreted in all inducing conditions, while only a few members were substrate-specific (Fig. 1A). The highest level of CAZymes secretion, estimated by the sum of normalized peptide counts, was observed in the presence of Avicel followed by SCB and SCS. Concerning the cultivation period, the trend was increasing secretion overtime in all tested conditions (Fig. 1B).

A total of 22 members were assigned to different AA families, numerically corresponding to the second most secreted class of CAZymes. The identified AAs corresponded to members from families AA2 (1), AA3 (5), AA7 (9), AA9 (6) and AA13 (1). Apart from AA9s (described below), most of the identified AAs were concomitantly secreted on the different substrates, excepting the AA2 catalase/peroxidase (AN7388) exclusively found on glucose, the AA13 LPMO (AN5463 appended to CBM20) secreted only on Avicel, and the AA7 dehydrogenase (AN2648) absent only in the glucose secretome. Among AA3s, one AA3_2 glucose oxidase (AN4006) was secreted on Avicel and SCB, one AA3_3 alcohol oxidase (AN0567) was exclusively found on SCB, and the AA3_1 CDH (AN7230 appended to CBM1) was present in all secretomes, excepting glucose (Table S1). Indeed, this CDH (*An*CDH1) was abundantly secreted, corresponding to 57, 34 and 30% of the total AA spectra on Avicel, SCB and SCS, respectively. While its secretion decreased overtime on Avicel, it was found at variable levels on SCB and SCS (Fig. 1C).

The set of LPMO9s, named according to Nekiunaite et al. (48) consists of *An*LPMO9A (AN2388), *An*LPMO9B (AN1602, appended to CBM1), *An*LPMO9C (AN6428), *An*LPMO9D (AN3046), *An*LPMO9F (AN3860) and *An*LPMO9G (AN10419). In contrast to the increasing profile of CAZymes secretion, the overall secretion of LPMO9s was generally more abundant in the early phase of cultivation, decreasing in a time-dependent manner (Fig. 1D). The secretion profiles on Avicel and SCS were quite similar both quantitatively and qualitatively, being mainly composed of *An*LPMO9F complemented by minor and constant amounts of *An*LPMO9G. Additionally, the SCS secretome included small amounts of *An*LPMO9D and *An*LPMO9C which were found at 24 and 72 h, respectively. In turn, SCB induced a more diversified secretome, which included the concomitant secretion of all the six LPMO9s at variable levels overtime. *An*LPMO9D was highly secreted at 24 h and decrease at late periods, whereas *An*LPMO9F was detected at a steady and intermediate level. The SCB repertoire also included the secretion of *An*LPMO9C that decreased overtime, *An*LPMO9G at intermediate-to-small amounts, and small amounts of *An*LPMO9A and *An*LPMO9B (exclusively found in the SCB secretome). Remarkably, small amounts of *An*LPMO9A were also identified on glucose at 24 and 72 h (Fig. 1D). Because of the *An*LPMO9F and -G production in all inducing conditions, and the exclusive secretion of *An*LPMO9C on lignocellulosic substrates, these three enzymes were selected for further functional studies.

### Target *An*LPMO9s are active on cellulose.

To confirm their oxidative activities, the genes encoding *An*LPMO9C, -F and -G were cloned with their native signal peptides and produced in A. nidulans by homologous expression. The purified recombinant enzymes showed higher MW (30.8, 29.5 and 33.4 kDa for *An*LPMO9C, -F and -G, respectively) than the predicted from amino acid sequence (22.5, 24.1 and 26.5 for *An*LPMO9C, -F and -G, respectively). The higher MW observed may be attributed to glycosylation, considering the presence of both N- and O-glycosylation sites predicted by NetNGlyc 1.0 Server and NetOGlyc 4.0 Server, respectively. According to the GLYCAM-Web tool, the *An*LPMO9C is the only target harboring accessible sites for N-glycosylation, and indeed the MW decreased by Endo H and PNGase F treatment (25.8 kDa - data not shown), confirming the presence of N-glycosylated residues. Correct processing of the purified enzymes was confirmed by determining the N-terminal sequence, which yielded sequences identical to those predicted from the corresponding nucleotide sequence (data not shown).

**FIG 2:**
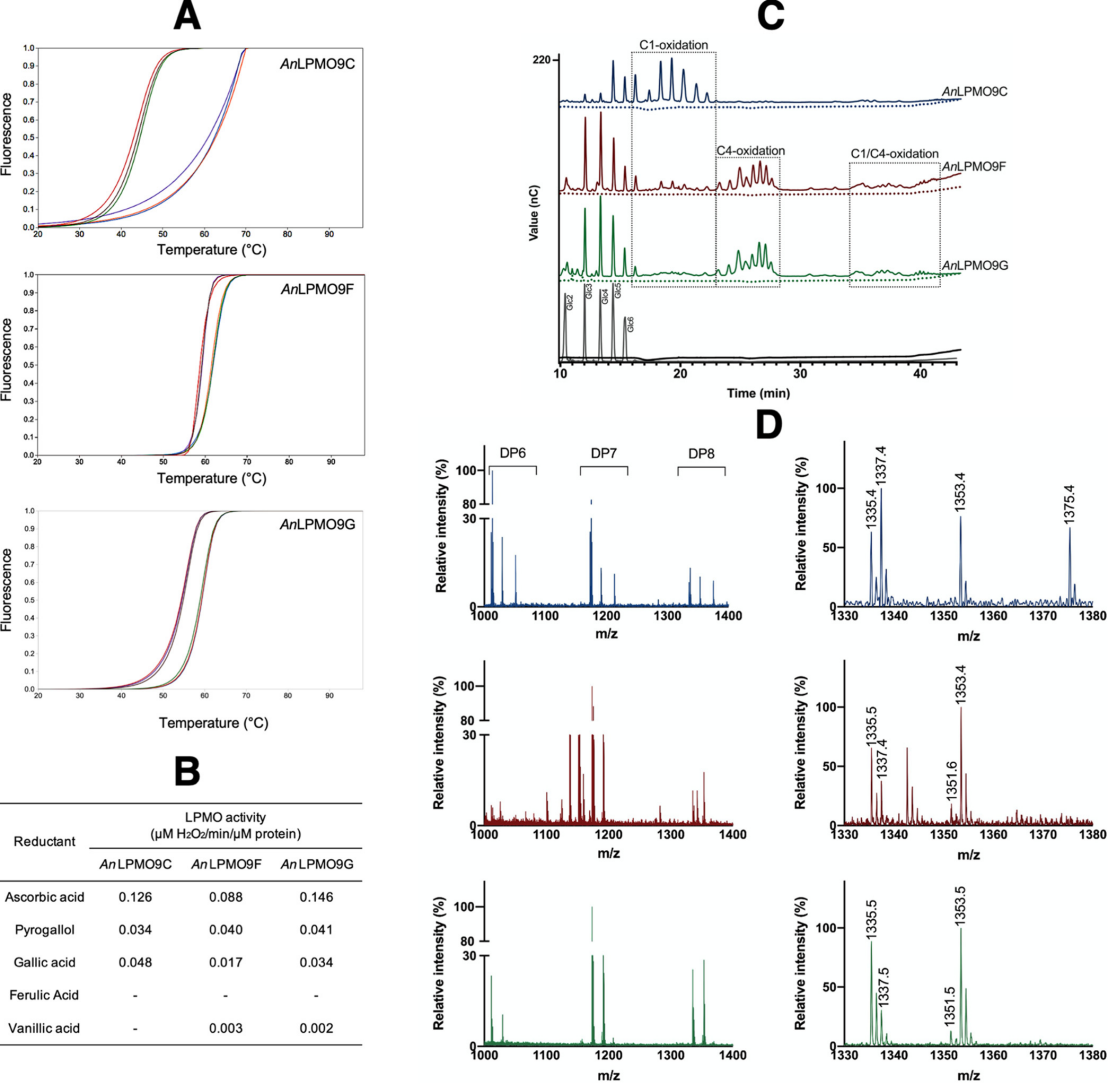
Characterization of recombinant A. nidulans LPMO9s. (A) DSF assays showing *T_m_* reductions upon copper ion removal of *An*LPMO9C (top), *An*LPMO9F (middle) and *An*LPMOG (bottom); (B) Amplex Red assays showing the response to different electron donors; (C) HPAEC-PAD chromatogram and (D) MALDI-TOF MS spectra of products generated from PASC reactions with *An*LPMO9C (blue), *An*LPMO9F (red) and *An*LPMOG (green). In (A), each set of colored lines represent technical replicates of copper-loaded (set on the right) and copper-deprived (set on the left) *An*LPMO9s. In (B), assays were performed as triplicate independent experiments and controls correspond to reactions with the copper-deprived *An*LPMO9s. The chromatogram (C) shows C1-oxidized cellooligosaccharides eluting at 16–23 min produced from treatment with the three *An*LPMO9s; at 23–28 min elution of diagnostic degradation products which are indicative of C-4 oxidized products released by *An*LPMO9F and -G; and at 34–42 min elution of double-oxidized (C1/C4) cello-oligosaccharides released by *An*LPMO9F and -G. Early eluting native cello-oligosaccharides (DP2-6) were more abundant in the *An*LPMO9F and -G chromatograms and may be partly attributed to technical on-column degradation of C4 oxidized species, in addition to true reaction products, which indeed are known to be more abundant in reactions performed by C1/C4-oxidizing LPMOs. Controls correspond to reactions without ascorbic acid (dotted lines) and a reaction without any enzyme (black line). Standards (gray line) correspond to Glc2–cellobiose, Glc3–cellotriose, Glc4–cellotetraose, Glc5–cellopentaose, Glc6–cellohexaose. (D) MS spectra show the presence of native and oxidized cello-oligosaccharides, as represented by the DP 6–8 clusters obtained for the three enzymes (on the left). DP8 close-ups (on the right) show mono-sodiated nonoxidized species (*m/z* 1337), and the oxidized forms corresponding to mono-sodiated lactone or ketoaldose (*m/z* 1335), and mono-sodiated aldonic acids or gemdiol species (*m/z* 1353). In addition, the di-sodiated aldonic acid form (*m/z* 1375), a hallmark of C1-oxidation, was present only in the *An*LPMO9C reactions, whereas double-oxidized sodium adducts (*m/z* 1351) were only observed in the *An*LPMO9F and -G reactions. Notably, certain amounts of unexpected products were observed in the *An*LPMO9F reactions, while absent in the reactions with *An*LPMO9C and -G. The origin and nature of these species remain unknown and may be arisen from imprecise binding of the substrate, generating nonspecific products.

Differential scanning fluorimetry (DSF) assays revealed higher stability (Tm of 60, 62, 59°C for *An*LPMO9C, -F and -G, respectively) of the holoenzymes (copper loaded) than the Cu-deprived enzymes obtained after incubation with EDTA (Tm of 44, 59, 55°C for *An*LPMO9C, -F and -G, respectively). The change in thermal stability confirmed the presence of copper coordinated in the active site of the enzymes providing stabilization to their structures (Fig. 2A). Enzymatic activity of the recombinant *An*LPMO9s was initially verified by Amplex Red assays, in which hydrogen peroxide formation indicates that the enzymes were active, therefore responding to the presence of different reductants. The highest activity for the three enzymes was observed using ascorbic acid (Fig. 2B).

Enzymatic assays with the *An*LPMO9s were then performed using phosphoric acid-swollen cellulose (PASC) as the substrate and ascorbic acid as external reductant. Analysis of reaction products from the three *An*LPMO9s by HPAEC-PAD and MALDI-TOF showed the presence of native and oxidized cellooligosaccharides. Taken together, these assays confirmed the activity and regioselectivity of each enzyme on cellulose, i.e., the C1-oxidation performed by *An*LPMO9C, and the mixed C1/C4-oxidation by *An*LPMO9F and -G (Fig. 2C and D). When similar enzymatic reactions were performed using Avicel the enzymes maintained their regioselectivity, but weaker signals were observed especially for *An*LPMO9C (Fig. S1A) in comparison to the reactions using PASC as the substrate (Fig. 2C). In turn, upon incubation with cellohexaose, beechwood xylan, or PASC/xylan mixtures, no oxidized products from cellohexaose or xylan were identified (data not shown).

**FIG 3:**
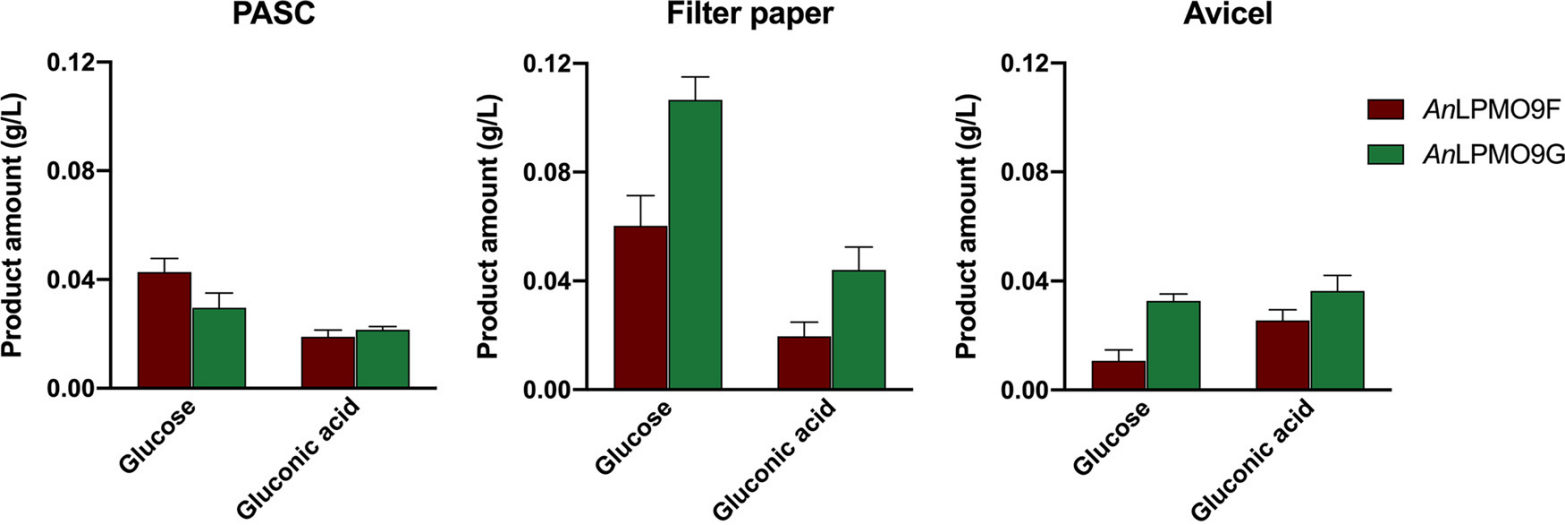
Products released from *An*LPMO9s acting on cellulosic substrates followed by β-glucosidase-assisted hydrolysis of soluble products. The soluble supernatant from *An*LPMO9F and -G reactions with PASC, filter paper and Avicel were hydrolyzed by a commercial β-glucosidase. Native and C1-oxidized glucooligosaccharides were degraded mainly into glucose and gluconic acid. HPAEC-PAD analysis was performed using glucose, gluconic acid and cellooligosaccharides commercial standards. Only trace amounts of cellobiose, cellotriose, and possibly cellobionic acid were detected and therefore not quantified. Control reactions without ascorbic acid and a reaction without any enzyme showed no released products. Note that the results are an estimate of mainly native and minor C1 oxidized species, as oxidation at the C4-position would hinder the action of the β-glucosidase.

To further investigate the catalytic performance of the *An*LPMO9s on cellulose, we used a β-glucosidase-assisted assay which allowed degradation of the soluble gluco-oligosaccharides released from reactions applying *An*LPMO9F and -G on PASC, filter paper (FP) and Avicel. The results correspond to an estimate of mainly native and minor C1 oxidized species, as oxidation at the C4-position possibly hinders β-glucosidase activity. Higher glucose amount was released from PASC using *An*LPMO9F, whereas using *An*LPMO9G more glucose was obtained from reactions with cellulosic substrates containing crystalline regions such as FP and Avicel (Fig. 3). In addition, activity was also evaluated on cellulose microcrystals extracted from the algae *Valonia ventricosa*, and only *An*LPMO9G was active (Fig. S1B).

### LPMO9s are important factors in the A. nidulans extracellular oxidative system, and their deletion reduce fungal growth on lignocellulose.

Based on the LPMO9s secretion profiles and the verified oxidative activity on cellulose, we hypothesized that *An*LPMO9F and -G, as well as the cosecreted *An*CDH1, would be important oxidative players for cellulose degradation by A. nidulans. To investigate the biological role of these enzymes in A. nidulans, knockout strains carrying single deletions, the respective double mutants as well as the triple mutant were built using CRISPR-Cas9 approach, and gene knockout was confirmed by PCR and Southern blot (Fig. S2).

**FIG 4:**
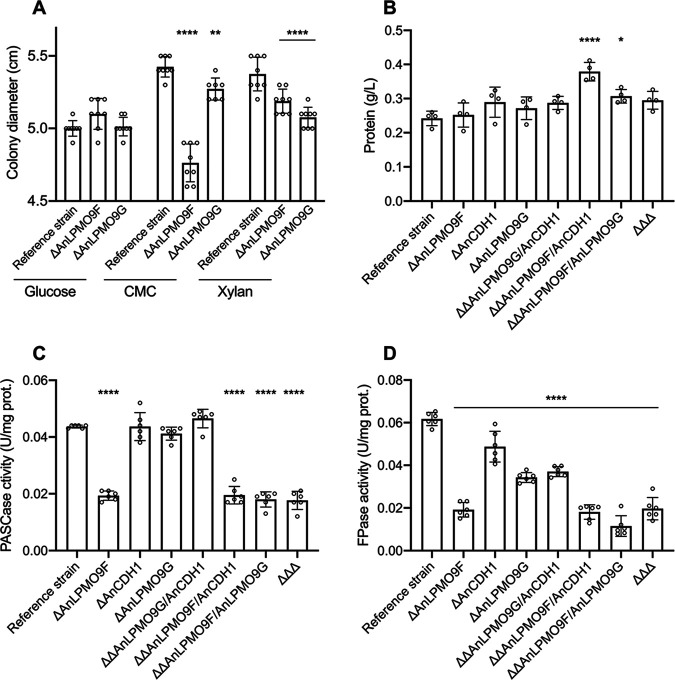
Characterization of A. nidulans mutants carrying single, double and triple deletions for *An*LPMO9F (AN3860), *An*LPMO9G (AN10419) and *An*CDH1 (AN7230). (A) Fungal growth on agar plates supplemented with glucose, CMC, and xylan as the substrate for 96 h; (B) protein secretion, (C) cellulolytic activity on PASC (PASCase), and (D) on filter paper (FPase) were measured in the secretome induced on Avicel. Error bars indicate standard deviations from six replicates. Statistics were taken from Dunnett's multiple comparisons used as follow up test to ANOVA. *, *P* < 0.05; **, *P* < 0.01; ***, *P* < 0.001; ****, *P* < 0.0001.

**FIG 5:**
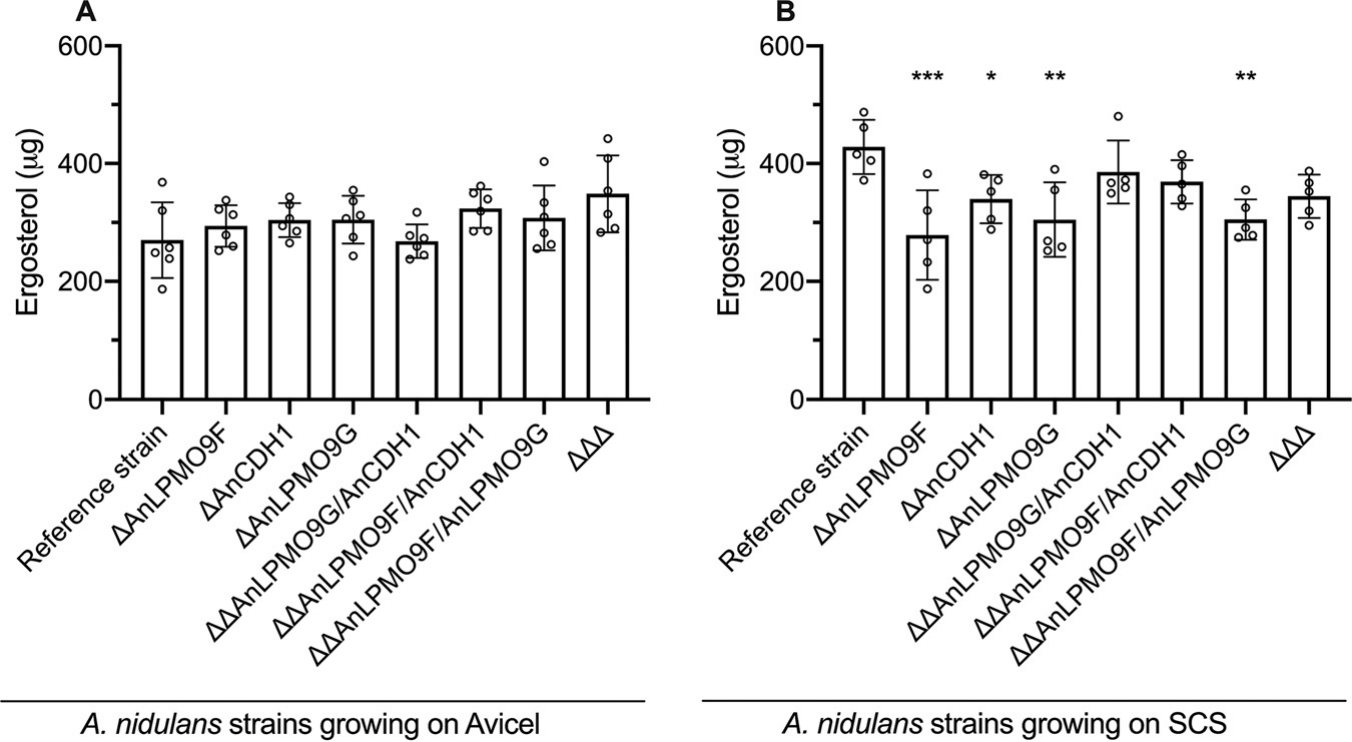
Growth analysis of A. nidulans mutants carrying single, double, and triple deletions for *An*LPMO9F (AN3860), *An*LPMO9G (AN10419) and *An*CDH1 (AN7230) cultivated on (A) Avicel and (B) SCS. Growth was indirectly measured by mycelial ergosterol quantification. Error bars indicate standard deviations from five or six replicates. Statistics were taken from Dunnett's multiple comparisons used as follow up test to ANOVA. *, *P* < 0.05; **, *P* < 0.01; ***, *P* < 0.001; ****, *P* < 0.0001.

Growth analysis on solid minimal medium (MM) supplemented with different substrates showed the mutant strains growing at the same rate on glucose, whereas reduced growth was verified for the single mutants Δ*An*LPMO9F and Δ*An*LPMO9G growing on cellulose and xylan, in comparison to the reference strain (Fig. 4A). The strains were then cultivated in liquid MM with Avicel, and the extracellular enzymatic activities profiles, as well as fungal growth, were investigated. Initial analysis revealed no changes in the secretory system since, overall, the secretomes presented similar levels of protein secretion relative to the reference strain (Fig. 4B). Deletion of the *An*LPMO9F had a major impact on the activity on PASC (PASCase), which was reduced by more than 60%. Additional deletions, however, had no further impact, i.e., the secretomes of the double knockout strains Δ*An*LPMO9F/Δ*An*LPMO9G and Δ*An*LPMO9F/Δ*An*CDH1, as well as the triple mutant, presented similar activity levels than the Δ*An*LPMO9F single mutant. Additionally, single and double deletions of *An*LPMO9G and *An*CDH1 had no impact on the activity on this substrate (Fig. 4C). Filter paper activity (FPase) was also strongly affected by *An*LPMO9F deletion, whereas single deletions of *An*LPMO9G and *An*CDH1 also reduced this activity at moderate levels. Double deletions of the *An*LPMO9s reduced FPase to the lowest level (Fig. 4D). Deletions were less influential for the degradation of hydroxyethyl cellulose (HEC) as the activities were partially reduced to the same level in all mutants; and the activity on β-glucan underwent reductions similar to that observed on PASC. Moreover, some mutants showed reduced activity on 4-nitrophenyl β-d-glucopyranoside (pNPG), xylan and 4-nitrophenyl β-d-xylopyranoside (pNPX), whereas most of the mutants displayed higher activity on 4-nitrophenyl β-d-cellobioside (pNPC) (Fig. S3). Despite the remarkable reductions in the extracellular cellulolytic activity of the knockout strains, no significant changes in fungal growth were verified when the strains were cultivated on Avicel (Fig. 5A). However, when cultivation was performed on SCS, deletions caused about 25% reduction in A. nidulans growth, of A. nidulans growth, as observed for the single and the double Δ*An*LPMO9F/Δ*An*LPMO9G mutants (Fig. 5B).

**FIG 6:**
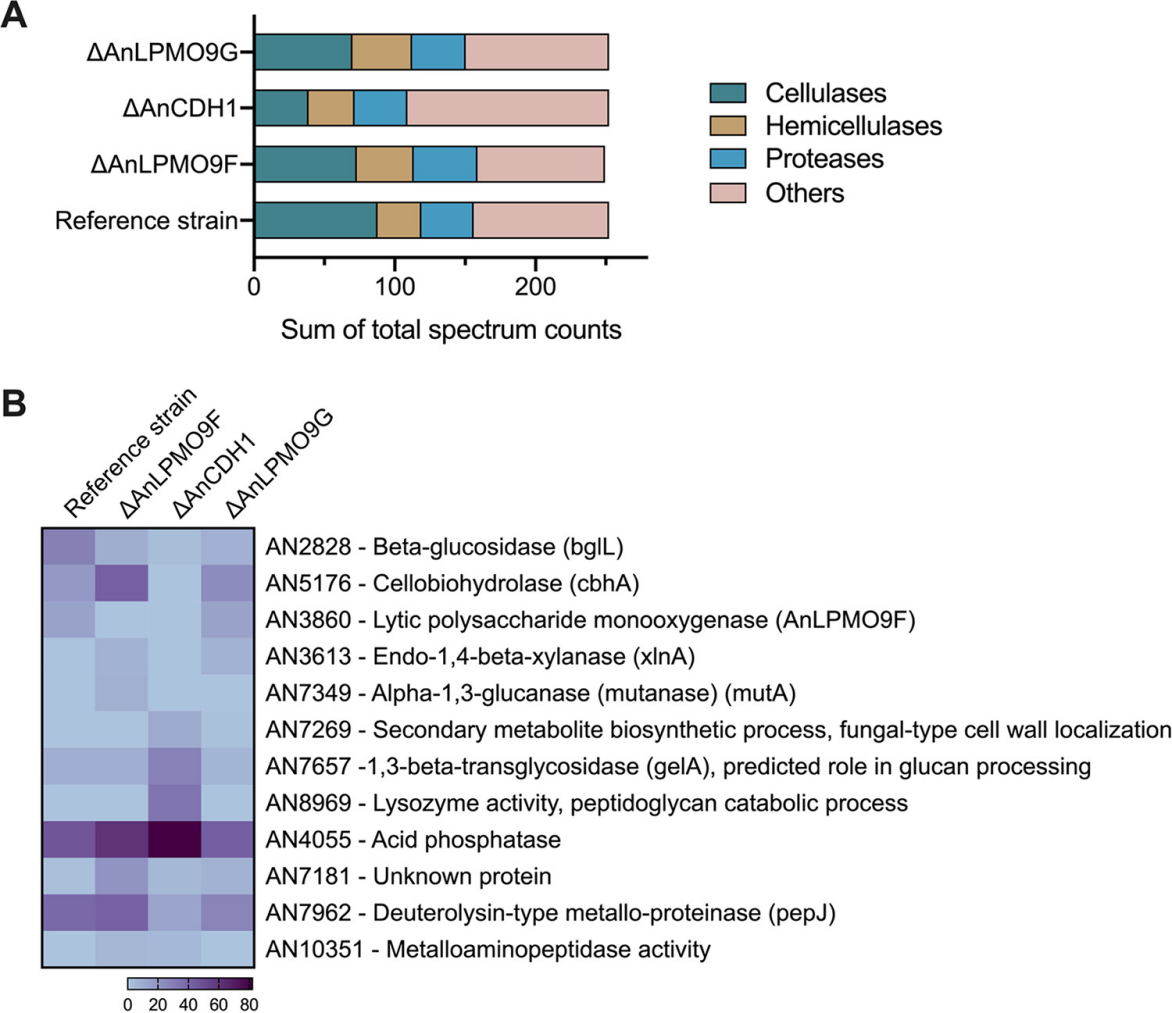
Analysis of proteins identified by MS in the extracellular proteomes of A. nidulans mutants carrying single deletions for *An*LPMO9F (AN3860), *An*LPMO9G (AN10419) and *An*CDH1 (AN7230) cultivated on Avicel. (A) Quantitative secretion analysis - abundance of identified proteins estimated by the sum of peptide counts from normalized data in comparison to the reference strain. Proteins were categorized as presenting activity on cellulose, on hemicellulose, proteases, and “others,” including miscellaneous enzymes and proteins of unknown function. (B) List of identified proteins with differential secretion (*P* < 0.05).

To further explore modifications in the microbial metabolism, the secretomes of the single deletion mutants induced on Avicel were analyzed by MS (Table S2). Overall, no changes in protein secretion were verified considering normalized peptide counts, confirming the data from the protein quantification assay. However, quantitatively a differential secretion was verified by categorizing enzymes in the following groups: cellulases, hemicellulases, proteases, and miscellaneous enzymes and proteins with unknown functions (named as “others”). Peptide counts of cellulases were reduced by around 20% in the LPMO9s knockout strains, whereas hemicellulases were approximately 30% more abundant (Fig. 6A). Particularly, changes in the secretion of β-glucosidase (*bglL*, AN2828) and cellobiohydrolase (*cbhA*, AN5176) can be related to the changes in the cellulolytic profile of the mutant strains, while secretion of xylanase (*xlnA*, AN3613), absent in the reference strain secretome, is a remarkable change in the hemicellulases group (Fig. 6B). Notably, in the Δ*An*CDH1 strain, peptide counts of cellulases were reduced by more than 50% and a striking increase occurred in the secretion of the category “others” (Fig. 6A), which included fungal cell wall-associated proteins (AN7269, AN7657, AN8969 and AN4055) (Fig. 6B).

### Remodeling A. nidulans secretomes by *An*LPMO9s overexpression.

**FIG 7:**
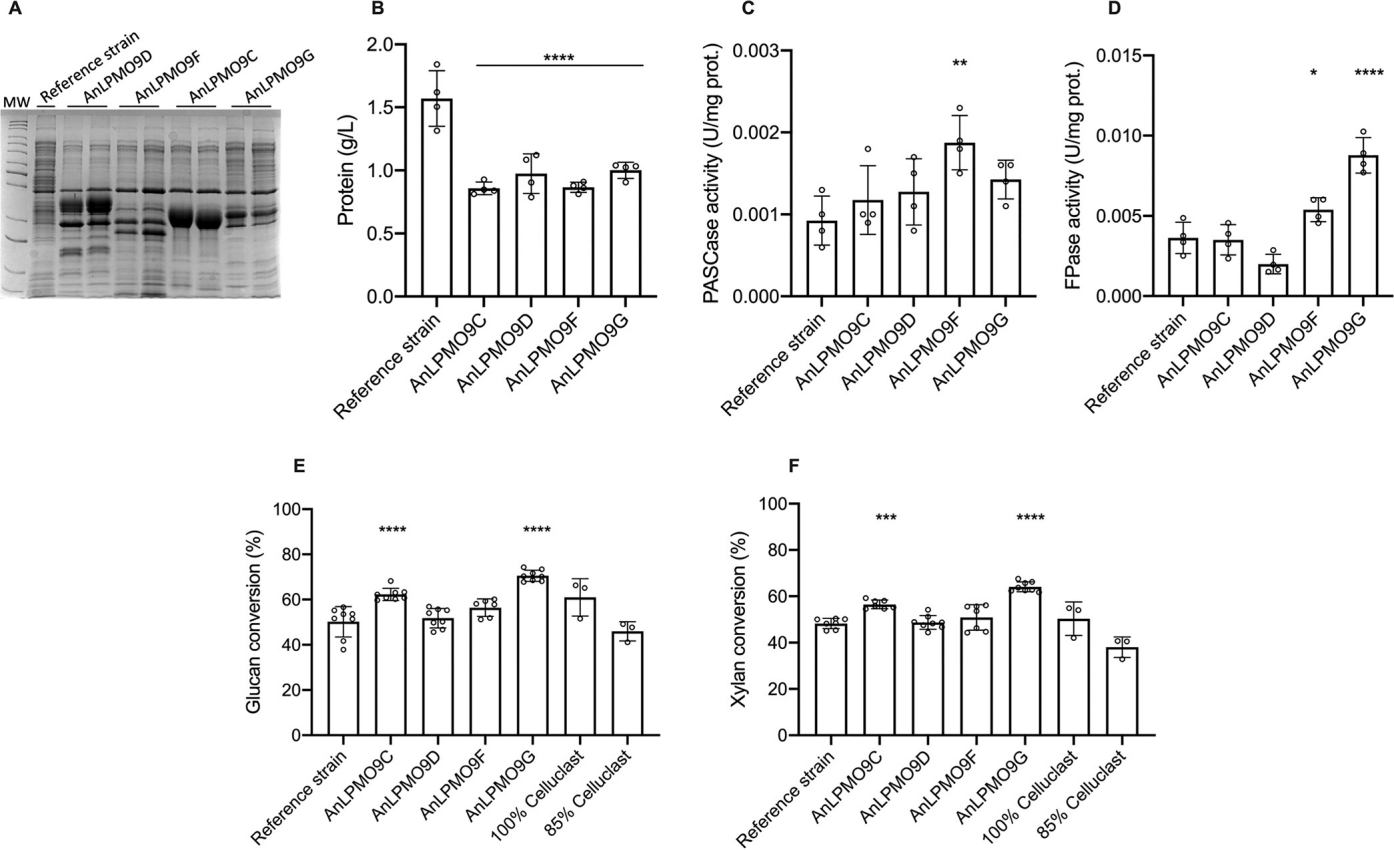
Characterization and application of the A. nidulans LPMO9-enriched secretomes. Co-expression of cellulolytic enzymes and high levels of the targets AnLPMO9s were achieved by the cultivation of A. nidulans strains overexpressing *An*LPMO9C (AN6428), *An*LPMO9D (AN3046), *An*LPMO9F (AN3860) and *An*LPMO9G (AN10419) in liquid MM supplemented with Avicel followed by maltose addition. (A) SDS-PAGE loaded with 20 μg of protein of two replicate secretomes. (B) Extracellular protein and cellulolytic activity using (C) PASC (PASCase) and (D) filter paper (FPase) as the substrates. (E) Glucan and (F) xylan conversion of SCS saccharification performed by the enriched secretomes supplementing commercial cocktails. Glucose, cellobiose and xylose were quantified by HPLC. Error bars indicate standard deviations from four replicates. Statistics were run against the reference strain secretome and taken from Dunnett's multiple comparisons used as follow up test to ANOVA. *, *P* < 0.05; **, *P* < 0.01; ***, *P* < 0.001; ****, *P* < 0.0001.

In contrast to gene deletion, we used A. nidulans recombinant strains overexpressing *An*LPMO9C, -D, -F and -G, aiming to increase the titer and/or change the LPMO9s profile relative to the originally verified on cellulose. The strategy adopted to obtain LPMO9s-enriched secretomes was initial cultivation on Avicel, a condition which naturally induces the secretion of *An*LPMO9F, *An*LPMO9G and *An*CDH1 along with several other cellulases (Fig. 1C and D, Table S1), followed by recombinant *An*LPMO9s induction by maltose. We evaluated a second strategy in which recombinant LPMO9s maltose-dependent induction was concomitant to the cultivation on Avicel (data not shown); however, the two-step strategy resulted in secretomes displaying higher levels of the target enzymes (Fig. 7A). The recombinant strains secreted lower levels of proteins (Fig. 7B) in comparison to the reference strain (transformed with the empty vector), and the enzymatic profiles using various substrates were distinct among them. In this regard, the *An*LPMO9F-enriched secretome was more active on PASC (Fig. 7C), whereas the *An*LPMO9G-enriched secretome presented increased FPAse activity (Fig. 7D). Variations on xylanase activity were also observed in the *An*LPMO9C, -F and -G-enriched secretomes. Notably, the *An*LPMO9D enriched secretome retained the same activity levels or underwent severe activity reductions (Fig. S4). When applied as supplementing agents for lignocellulose saccharification, the secretomes enriched with *An*LPMO9C and -G demonstrated to increase both glucan and xylan conversions. Remarkably, supplementation with the *An*LPMO9G-enriched secretome resulted in glucan conversion yields superior to that of using total enzyme load made up of the commercial cocktail (Fig. 7E and F).

## DISCUSSION

Among several CAZymes differentially secreted, six *An*LPMO9s had their secretion influenced by the inducing substrate and period of cultivation. The profile of LPMO9s induced in the presence of Avicel or SCS were quite similar, but two additional members were identified in the SCS secretome at small amounts, probably induced by lignocellulosic components other than cellulose or cellooligosaccharides. In turn, SCB induces a more diverse set of LPMO9s, which can be associated with the distinct compositional contents of SCB and SCS (49) as well as hemicellulose (50) and lignin (51) structures, both of which parameters can be affected by the plant biomass pretreatment (52). The secretion levels of these enzymes are remarkably high at the early period of cultivation, especially on SCB, decreasing overtime in all cultivation conditions. Likewise, when a different lignocellulosic feedstock such as sorghum stover is used for A. nidulans cultivation a similar profile is obtained, considering both the *An*LPMO9s variety and time response (2). In addition to the induction by complex lignocellulosic substrates, it is notable that some *An*LPMO9s can also be induced by noncellulosic substrates such as starch (48), commercial xylan and glucose (2). Despite the limited number of studies involving the regulation of LPMO9s expression in fungi, several binding sites for common CAZymes regulators such as CreA, CeRE, and XlnR can be found in promoter regions of the *An*LPMO9s encoding genes (25) and the expression of *An*LPMO9C, -F and -G showed to be strongly dependent on CLR-B, another transcription factor involved in the regulation of cellulases (53, 54).

The abundant secretion of *An*CDH1, highly induced on Avicel, is an indication that this enzyme plays an important role in the A. nidulans oxidative system. In turn, the lower secretion levels on SCS and SCB suggest the presence of alternative electron donors such as residual lignin or its derived compounds found in plant biomass (18, 55–58) as well as other enzymes (discussed below). In addition to a strong induction by crystalline cellulose (25, 53, 54), other studies detected *An*CDH1 expression/secretion also occurring on different lignocelluloses, but not on xylan (5, 25), evidencing it is specifically induced by cellulose or cellulose-derivatives.

Recombinant *An*LPMO9C, -F and -G were abundantly secreted in their active forms using a cloning strategy widely used for CAZyme expression in filamentous fungi (59). As predicted by phylogeny (25), our functional analysis demonstrated that these LPMO9s are cellulose-active enzymes with different regioselectivities. *An*LPMO9F and -G found in the secretomes induced on Avicel, SCB and SCS oxidize C1 and C4 in glycosidic linkages, whereas *An*LPMO9C, detected only in the secretomes induced on lignocellulosic feedstocks, oxidizes C1. These newly characterized enzymes thereby expand the current knowledge on the *An*LPMO9s arsenal in addition to the previously characterized *An*LPMO9D (AN3046), which oxidizes at C1 of cellulose and xyloglucan (25) and *An*LPMO9B (AN1602, appended to CBM1), which oxidizes at C4 of cellulose and cello-oligosaccharides (47). A. nidulans takes place along with other fungi such as N. crassa, *Gloeophyllum trabeum*, *Malbranchea cinnamomea,* and *P. anserina*, which have the LPMO9 arsenal extensively characterized (15).

While the three enzymes were active on cellulose, a comparison between *An*LPMO9F and -G demonstrated that, ultimately, the latter showed higher performance on celluloses that have crystalline regions such as Avicel and FP which presents 30–50% of amorphous regions (60–62). Additionally, only *An*LPMO9G was active on the *Valonia* cellulose which corresponds to one of the most crystalline native cellulose materials (63–66).

Based on the secretion profiles on Avicel and SCS, single, double, or triple mutants carrying deletions of *An*LPMO9F, *An*LPMO9G and *An*CDH1 were designed to verify their contribution to the A. nidulans cellulolytic system. Deletion of the most secreted *An*LPMO9F (single, double, or triple mutants) had a striking impact, reducing by a half the activity toward amorphous cellulose or β-glucan. In contrast, these activities were not affected by the *An*LPMO9G deletion. In turn, when FP was used as the substrate, single deletions of both *An*LPMO9s had a negative effect on the enzymatic activity. Considering these data, as well as the minor and steady secretion of *An*LPMO9G, it is likely that this enzyme oxidizes crystalline portions of the substrate, while the abundantly secreted *An*LPMO9F appears more efficient on cellulose with a lower degree of crystallinity or a higher extent of amorphous regions. Moreover, the overexpression of *An*LPMO9F and -G improves the secretome activity on PASC and FP, respectively.

The CDHs and LPMOs interplay in the fungal breakdown of crystalline cellulose has been demonstrated *in vitro* (31–35). Here, our *in vivo* study shows that despite the high secretion levels of *An*CDH1 under induction by cellulose, the performance of the Δ*An*CDH1 strain secretome is not changed using amorphous cellulose as the substrate. Only a subtle decrease can be verified in the FPase, indicating that *An*CDH1 may also contribute to the degradation of crystalline cellulose. Additionally, its deletion causes no impairment of fungal growth on crystalline cellulose and a subtle decrease on lignocellulose. While there are no such studies involving LPMO9s, some functional studies dealing with CDH deletion have been published in the last few years reporting variable results obtained with different fungal species. In *T*. *versicolor*, CDH deficient mutants grew poorly in minimal medium supplemented with crystalline cellulose, but not on noncrystalline carbohydrates. In addition, these mutants are deficient in colonizing and degrading wood (36). In N. crassa, deletion of the major CDH substantially reduced the secretome activity on crystalline cellulose (33). In *P*. *anserina*, single and multiple mutants lacking CDHs can grow normally on cellulose, but some mutants present reduced conidia formation, possibly due to a reduced ability to obtain nutrients from crystalline cellulose. Besides, external LPMO9s supplementation can improve the performance to degrade cellulose using the parental or the CDH deficient secretomes, suggesting that other redox partners are providing electrons to the *P. anserina* LPMO9s (38). Indeed, *in vitro* analysis have been revealing other enzymes, such as AA3_2 flavoenzymes (67), AA12 pyranose dehydrogenase (68), and AA7 oligosaccharide dehydrogenase (69), acting as electron donors to the LPMO9s. Here, A. nidulans AA7 or AA3 oxidoreductases were exclusively secreted on lignocellulose and Avicel, representing potential alternative redox partners for the *An*LPMO9s.

Other changes in the secretomes such as the reduction in the xylanolytic activity could not be easily explained since the target *An*LPMO9s showed no oxidative activity on isolated xylan or cellulose/xylan mixtures, and the MS analysis of the single-deleted mutants showed increased secretion of xylanolytic enzymes. Additionally, only a few LPMO9s are known to display activity on hemicelluloses and oligosaccharides, which usually occurs in addition to the activity on cellulose (70), or, more rarely, only on substrate mixtures (71).

MS analysis of secretomes from the single deletion mutants also revealed an overall reduction in cellulase secretion, particularly of a β-glucosidase, corroborating the enzymatic assays (except for the Δ*An*LPMO9F strain). Furthermore, the strains lacking *An*LPMO9s increased the secretion of a cellobiohydrolase, suggesting an adaptive mechanism adopted to compensate LPMO9s absence during cellulose degradation. A compensatory mechanism is also observed in *P. anserina* mutants lacking CDHs, which secretes an increased number of β-glucosidases probably alleviating CBH inhibition by cellobiose, which is originally performed by CDH through product oxidation (38). However, this compensation mechanism by increasing β-glucosidase secretion was not observed in the A. nidulans mutant strains lacking LPMO9s or CDH. In addition, the increased secretion of proteins associated with fungal cell wall verified in the Δ*An*CDH1 strain suggests more intense cell-wall remodeling.

In summary, we identified several AA9 LPMOs as well as a CDH being differentially produced by A. nidulans upon growth on crystalline cellulose and lignocelluloses. One C1/C4-oxidizing LPMO9 (*An*LPMO9F) is predominant in the secretome induced on cellulose and sugarcane straw, while another C1/C4-oxidizing LPMO9 (*An*LPMO9G) is steadily secreted at small amounts in all inducing conditions. In turn, a more diversified set including several LPMO9s with distinct regioselectivities is induced on sugarcane bagasse. The phenotyping of A. nidulans mutant strains allowed measuring the importance of those oxidative components for the extracellular cellulolytic system, demonstrating that the lack of LPMO9s partially reduces fungal growth on lignocellulose. In turn, the impact of CDH absence was less evident, despite the high secretion levels under inducing conditions. Furthermore, the overexpression of specific LPMO9s also contributed to revealing the importance of each component in plant biomass degradation and gave rise to an enriched fungal secretome that boosts lignocellulose conversion when added to commercial enzymes.

## MATERIALS AND METHODS

### Microbial strains and growth conditions.

A. nidulans A773 (*pyrG89*; *wA3*; *pyroA4*; *veA1*) was obtained from the Fungal Genetic Stock Center (FGSC, University of Missouri, Kansas City, MO, USA) and used to generate A. nidulans Δ*ku* (*nkuA*/AN7753). The A. nidulans strains constructed in this project (Table S3) corresponded to those homologously expressing *An*LPMO9C, *An*LPMO9D, *An*LPMO9F and *An*LPMO9G and the single, double, and triple knockouts for *An*LPMO9F, *An*LPMO9G and *An*CDH1. These strains were routinely maintained on minimal medium (MM) composed of 20× Clutterbuck salts (72), 1000× pyridoxine, and 1000× trace elements, pH 6.5 supplemented with 1% (wt/vol) glucose and cultivated at 37°C. Parental strains were cultivated in the same medium, including 5 mM uracil and uridine. Escherichia coli DH5α was used to propagate all plasmids.

### Analysis of extracellular proteomes by mass spectrometry.

A. nidulans A773 conidia suspension were inoculated (5 × 10^6^ conidia/ml final concentration) for precultivation on Erlenmeyer flasks (250 mL) containing 50 mL liquid MM 1% glucose (wt/vol) pH 6.5 for 24 h at 37°C. Mycelia were removed by filtration, washed abundantly and transferred to Erlenmeyer flasks (250 mL) containing 50 mL liquid MM pH 6.5 supplemented with 1% (wt/vol) Avicel, glucose, steam-exploded sugarcane bagasse (SCB) and steam-exploded sugarcane straw (SCS), cultivation then proceeded for 24, 72 and 120 h at 37°C. Cultivation was performed using three replicates. Mycelia were removed by filtration and the crude filtrates were centrifuged (10.000*g*, 40 min, 4°C) and concentrated in 10 kDa cutoff Amicon (Millipore, USA). The concentrated secretomes (20 μg) were partially resolved on 12% SDS-PAGE, excised, reduced, and digested with 20 mg/ml trypsin (Promega) (73). After extraction, samples were dried under vacuum, peptide mixtures were analyzed by LC-MS/MS -LTQ Orbitrap Velos (Thermo Fisher Scientific), and data were analyzed as previously described (5).

### Cloning and expression of selected *An*LPMO9s.

The full-length ORFs encoding the targets *An*LPMO9C (AN6428), *An*LPMO9D (AN3046), *An*LPMO9F (AN3860) and *An*LPMO9G (AN10419) were PCR amplified from A. nidulans A773 gDNA using Phusion DNA polymerase, according to standard protocols and primers listed in Table S4. Amplicons were ligated into the pEXPYR expression vector (59) using BbvCI and XbaI restriction sites and transformed into E. coli DH5α. After cloning and confirmation by sequencing, the vector was transformed into A. nidulans A773 protoplasts (74). Transformants growing in the absence of uracil and uridine were screened for target enzyme secretion by cultivation in liquid MM supplemented with 1% (wt/vol) glucose and 2% (wt/vol) maltose, under static conditions for 36 h at 37°C. Cultivation supernatants were concentrated using 10 kDa cutoff Amicon (Millipore, USA) and protein profiles were analyzed by SDS-PAGE.

### Production and purification of recombinant *An*LPMO9s.

A. nidulans transformants expressing *An*LPMO9C, *An*LPMO9F and *An*LPMO9G were grown in Erlenmeyer flasks (2 L) containing 200 mL MM pH 6.5 supplemented with 1% (wt/vol) glucose and 2% (wt/vol) maltose. Cultivation was performed for 36 h under static conditions at 37°C. Cultivation supernatants were centrifuged (5.000*g*, 40 min, 4°C), filtered (0.45 um) and loaded onto a HiPrep DEAE FF 16/10 column (GE Healthcare), equilibrated in 50 mM Tris–HCl buffer pH 7.0. Proteins were eluted using a linear gradient up to 1 M NaCl in the same buffer. Fractions containing a protein band with the expected theoretical MW were pooled and concentrated. The concentrated sample was applied to a HiTrap Phenyl HP 1 mL column (GE Healthcare) and eluted in the same buffer with a linear gradient from 1.5 – 0.0 M ammonium sulfate. Fractions containing a protein band with the expected theoretical MW were pooled. Partially purified enzymes were incubated with Cu(II)SO_4_ (3:1 molar ratio of copper:enzyme) overnight at 4°C. Then, samples were concentrated, washed in 50 mM MES buffer pH 6.0 and applied to a Superdex 75 column (GE Healthcare) equilibrated in the same buffer. Eluted fractions were analyzed in SDS-PAGE and those presenting the purified enzyme were pooled. Protein concentration was determined by reading A_280_ and considering the theoretical MW and molar extinction coefficient of each enzyme.

### Characterization of recombinant *An*LPMO9s.

Differential scanning fluorimetry (DSF) with SYPRO Orange fluorescent dye (Sigma-Aldrich) was used to monitor protein unfolding (75). Reactions (30 μL) were composed of 25 mM Tris HCl buffer pH 7.0, 30 μg of purified *An*LPMO9C, *An*LPMO9F and *An*LPMO9G and 5-fold diluted SYPRO Orange. Stabilization provided by copper-binding to the LPMOs was determined by comparing their temperature of melting (T_m_) to the controls containing the enzymes previously incubated with 5 mM EDTA. Assays were performed as triplicate independent experiments. The T_m_ was determined by plotting fluorescence against temperature and fitting the data to a sigmoid function (76). The influence of reductants on the activity of *An*LPMO9C, *An*LPMO9F and *An*LPMO9G was verified with Amplex Red assays by measuring peroxide formation (77). The evaluated reductants were 50 μM ascorbate, pyrogallol, hydroquinone, ferulic acid, 2,6-dimethoxyphenol (2,6-DMP) and gallic acid. Assays were performed as triplicate independent experiments and controls correspond to reactions with the copper-deprived *An*LPMO9s. Enzymatic reactions with substrates were carried out in 50 mM sodium acetate buffer pH 5.0 containing 0.1% (wt/vol) PASC, 0.2% (wt/vol) beechwood xylan (Sigma-Aldrich), 0.2% (wt/vol) PASC/xylan mixture, 0.1% (wt/vol) *Valonia* cellulose, and 50 μM cellohexaose, 1.0 μM *An*LPMO9C, *An*LPMO9F and *An*LPMO9G and 1 mM ascorbate as reductant. PASC was prepared from Avicel PH-101 (Sigma-Aldrich) (78). *Valonia* cellulose microcrystals were prepared from vesicles of the algae *Valonia ventricosa* (64). Reaction mixtures were shaken in a ThermoMixer (Eppendorf) at 800 rpm and 50°C for 24 h. Enzymatic activity was stopped by heating the samples at 100°C for 5 min, soluble fractions were separated by centrifugation (13,000*g*, 5 min, 4°C) and stored at 4°C. All assays were performed as triplicate independent experiments and controls correspond to reactions without ascorbic acid and a reaction without any enzyme.

### Detection of oxidized oligosaccharides.

Oligosaccharides released from reactions with PASC were initially analyzed by high-performance anion-exchange chromatography (HPAEC) on a Dionex ICS5000 system (Thermo Fisher Scientific) equipped with a CarboPac PA1 14 mm column and pulsed amperometric detection (PAD) (79). Elution and identification of native and oxidized products were based on previously published data (80). Additionally, products from reactions with PASC, *Valonia* cellulose, cellohexaose, xylan and PASC/xylan mixture were analyzed on Ultraflex MALDI TOF mass spectrometer (Bruker Daltonics) (75). Spectra intensity (V) was expressed relative to the maximum values.

### Activity on cellulosic substrates.

β-glucosidase-assisted assay (81) was performed by incubating 2% (wt/vol) Avicel, FP and PASC in 50 mM ammonium acetate buffer pH 5.0 with 1 μM *An*LPMO9F and -G, and 1 mM ascorbic acid. The reactions were carried out under 800 rpm shaking in a ThermoMixer (Eppendorf) at 30°C for 24 h. Samples were centrifuged (13,300*g*, 15 min, 4°C), filtered (0.22 μm), and 450 μL of the supernatant was mixed with 1 U of commercial β-glucosidase from Aspergillus niger (Megazyme). The reactions were then incubated for 24 h at 40°C. Samples were cooled, diluted, and the products quantified using high-performance anion-exchange chromatography (HPAEC-PAD) (82). The experiment was performed with two replicates and controls corresponded to reactions without enzyme and reactions without ascorbic acid.

### Deletion of LPMO9s and CDH encoding genes.

CRISPR/Cas9 systems were constructed (83, 84) using primers (Table S4) to amplify noncoding regions flanking the targets AN3860 (*An*LPMO9F), AN10419 (*An*LPMO9G) and AN7230 (*An*CDH1) on the A. nidulans genome. Constructed vectors and synthesized repairing oligonucleotides composed of upstream and downstream sequences of the target genes (Table S4) were used for transformation using A. nidulans Δ*ku* protoplasts. Transformants growing in the absence of uracil and uridine were submitted to five rounds of monosporic purification. Double and triple knockout strains were obtained by performing sequential rounds of deletion after having a confirmed mutant. Deletions were confirmed by diagnostic PCR and Southern blot (85) using genomic DNA of the knockout strains.

### Characterization of the knockout mutants.

For growth analysis in solid medium, conidia suspensions of the knockout strains were inoculated on the center of a petri dish containing MM pH 6.5 supplemented with 1% (wt/vol) glucose, carboxymethyl cellulose (CMC) and xylan from beechwood. Cultivation was performed with 5 replicates at 37°C and colony diameters were measured after 96 h. For liquid medium cultivation, conidia of the knockout strains (5 × 10^6^ conidia/ml final concentration) were precultivated in MM with 1% (wt/vol) glucose for 24 h and then mycelia were transferred for cultivation in MM Avicel for additional 48 h, as described before. The mixtures of mycelia and substrate were separated by filtration, manually dried with filter paper and stored at −80°C. The crude filtrates were centrifuged (10.000*g*, 40 min, 4°C), filtered (0.45 μm), concentrated when necessary (10 kDa cutoff Amicon) and used for protein and activity assays. Cultivation was also performed using 1% SCS (wt/vol) as the substrate at the same conditions. The solid fractions (Avicel and mycelia, and SCS and mycelia) were used for ergosterol extraction and quantification, as described below. Cultivation was performed using six replicates. Enzymatic activities in the secretomes were assayed using different substrates: 5 mM 4-nitrophenyl β-d-cellobioside (pNPC), 4-nitrophenyl β-d-xylopyranoside (pNPX) and 4-nitrophenyl β-d-glucopyranoside (pNPG) and 0.5% (wt/vol), carboxymethyl cellulose (CMC), hydroxyethylcellulose (HEC), xylan from beechwood (Sigma-Aldrich), β-glucan from barley (Megazyme). PASC was used at 1% (wt/vol); and FPAse assays were performed as previously described (86, 87). Reactions were performed with 1 – 20 μg of protein from the concentrated secretomes in 50 mM sodium acetate buffer pH 5.5, 50°C, during 0.5 – 1 h. Reactions with polymeric substrates were interrupted with 100 μL of DNS and the released reducing sugars were measured at 540 nm (88). Reactions with the synthetic substrates were interrupted with 100 μL of 1 M sodium bicarbonate and the released 4-nitrophenolate was measured at 405 nm. All assays were performed in triplicate. One enzyme unit (1 U) corresponds to the amount of enzyme that catalyzes the conversion of one μmol of substrate per minute under the assay conditions. The solid fraction (mycelia and substrate) of cultivations with Avicel and SCS were macerated with a mixture of 0.5 mm and 4 mm glass beads and 20% (wt/vol) KOH in methanol under vigorous vortexing for 2 min. The slurries were sonicated for 30 min and then incubated in a water bath at 65 – 70°C for 1.5 h. After cooling, water was added and ergosterol was extracted twice with hexane. Hexane was evaporated and ergosterol was recovered in methanol under sonication. Diluted samples (50 μL) were applied in an HPLC system equipped with A_280_ detector coupled to a C18 reversed-phase column (Supelco, 25 cm x 4.6 mm, 10 μm) and isocratically eluted with 1 mL/min methanol. Total ergosterol in the samples was quantified based on peak area using standard ergosterol (Sigma).

### MS analysis of single knockout secretomes.

The concentrated secretomes (20 μg) of the mutants carrying single deletions of *An*LPMO9F, *An*LPMO9G and *An*CDH1 (cultivated in Avicel for 24 h) were prepared from in-gel tryptic digestion, as described above. Digested peptides were separated using RP-nanoUPLC (nanoAcquity, Waters) on a C18 column coupled to a Q-TOF Premier mass spectrometer (Waters) with a nanoelectrospray ion source (89) and data analysis was performed as previously described (5).

### Enrichment of the secretome produced on Avicel with recombinant *An*LPMO9s.

Conidia suspensions (1 × 10^6^ conidia/ml final concentration) of the A. nidulans recombinant strains expressing *An*LPMO9C, *An*LPMO9D, *An*LPMO9F and *An*LPMO9G were precultivated in liquid MM with glucose, as described above. Mycelia was removed by filtration, washed with distilled water and transferred to Erlenmeyer flasks (250 mL) containing 50 mL liquid MM pH 6.5 with 1% (wt/vol) Avicel. After cultivation for 24 h, 180 rpm, 37°C, maltose was added at 2% (wt/vol) final concentration and cultivation proceeded for additional 24 h at the same conditions. Alternatively, induction by maltose was performed concomitantly to the cultivation in Avicel in 24 and 48 h cultivations at the same conditions. Cultivation was performed using four replicates. The crude filtrates were centrifuged (10.000*g*, 40 min, 4°C), filtered (0.45 μm), concentrated when necessary, (10 kDa cutoff Amicon) and used for protein and activity assays (as described above), and for lignocellulose saccharification.

### Lignocellulose saccharification.

The *An*LPMO9-enriched secretomes were evaluated by replacing 15% of protein load from commercial enzymes Celluclast (Novozymes) + glucosidase from Aspergillus niger (Merck) in the SCS degradation (90), following previously established reaction conditions (91). The products were analyzed by high-performance liquid chromatography (HPLC) in a 1260 Infinity system (Agilent) equipped with a refractive index (RI) detector coupled to an Aminex HPX 87H column (300 mm x 7.8 mm) at 35°C, 0.6 mL/min^−1^ flow rate and 5 mM sulfuric acid isocratic elution. Glucose, cellobiose and xylose were quantified by external calibration.

### Protein analysis.

Proteins were quantified by the BCA method (Thermo Scientific) and visualized in 12.5% SDS-PAGE (92) after staining with Coomassie blue R-25.

### Data availability.

The mass spectrometry proteomics data of A. nidulans A773 growing in different substrates at different periods of cultivation have been deposited to the ProteomeXchange Consortium via the PRIDE (93) partner repository http://proteomecentral.proteomexchange.org/cgi/GetDataset with the data set identifier PXD031886; and data of A. nidulans knockout mutants growing in Avicel with the identifier PXD031881.
