# Supplementary material for: Deletion of AA9 Lytic Polysaccharide Monooxygenases Impacts A. nidulans Secretome and Growth on Lignocellulose
Source: Microbiol Spectr. 2022 Jun 6;10(3):e02125-21. doi: 10.1128/spectrum.02125-21 (PMC9241910; doi:10.1128/spectrum.02125-21)
Supplement: Supplemental file 5 — Fig. S1-S4. Download spectrum.02125-21-s0005.pdf, PDF file, 0.8 MB [file spectrum.02125-21-s0005.pdf]

# Supplemental Figures

**A**

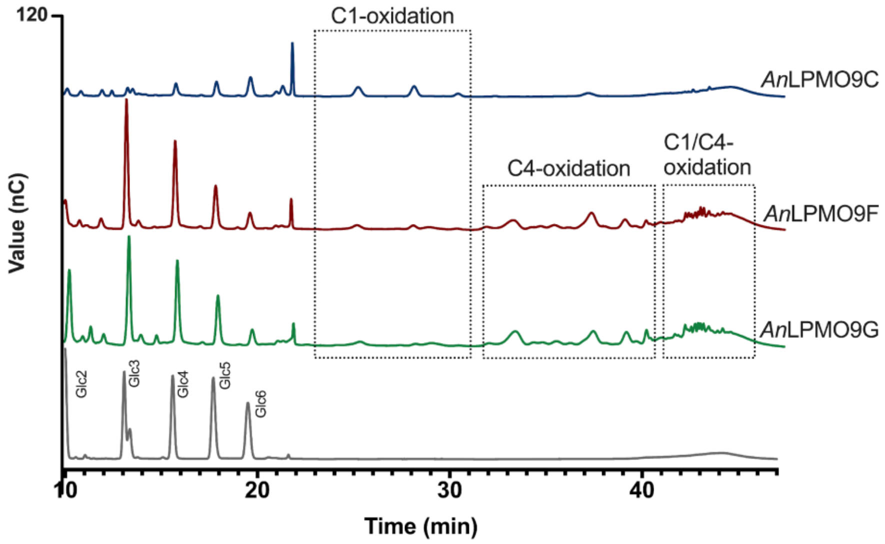


**B**

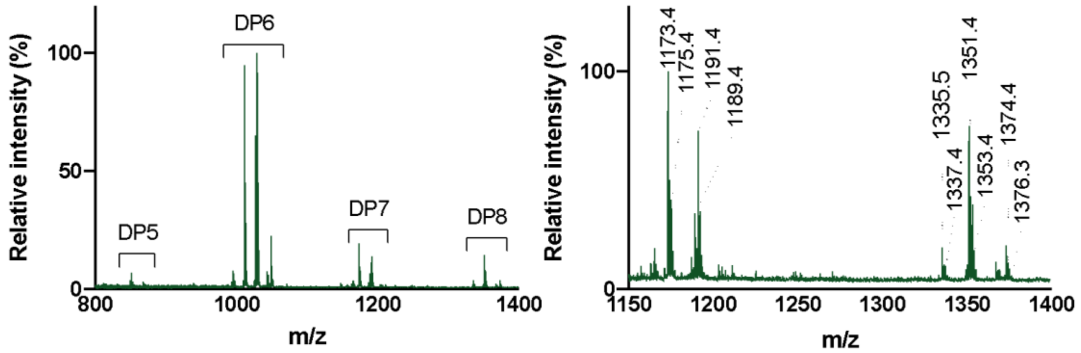


**Fig S1** Characterization of *An*LPMO9s. (A) HPAEC-PAD chromatogram of products generated from Avicel reactions with *An*LPMO9C (blue), *An*LPMO9F (red) and *An*LPMOG (green). (B) MALDI-TOF MS spectrum of products generated from *Valonia ventricosa* cellulose microcrystals reactions with *An*LPMOG (green). For details, refer to the legend of FIG 2 and Materials and Methods section.

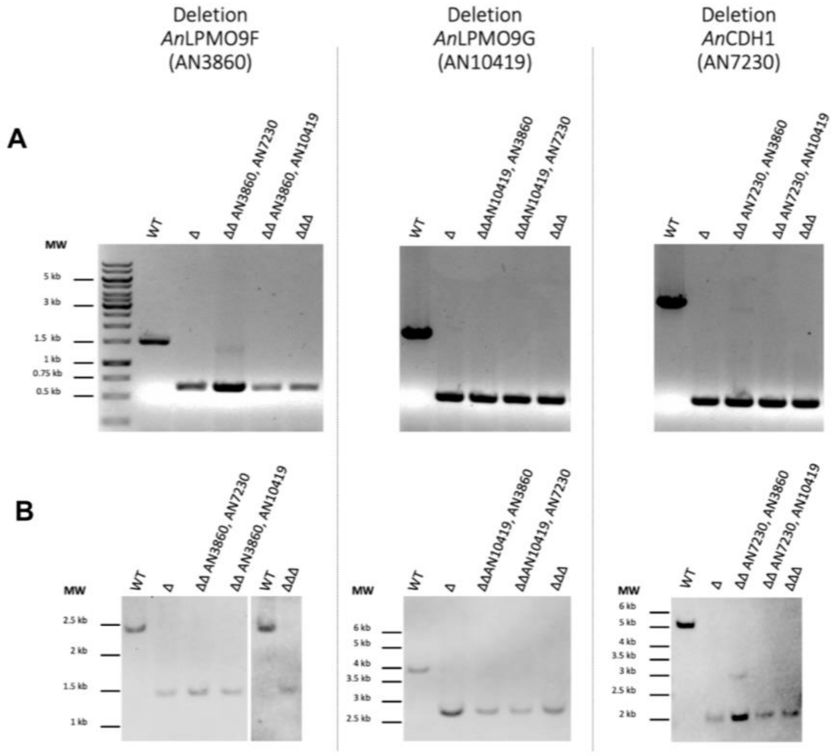


**FIG S2** Deletion of *An*LPMO9F (AN3860), *An*LPMO9G (AN10419) and *An*CDH1 (AN7230) in *A. nidulans* Δ*ku* strain using the CRISPR/Cas9 system. Deletion of each target gene was confirmed by performing (A) diagnostic PCR and (B) Southern Blotting using genomic DNA (gDNA) of each mutant. Agarose gel electrophoresis (above) shows the PCR products using the following templates: WT - gDNA from the parental strain *A. nidulans* Δ*ku* and gDNA from each derived knockout mutant. Expected sizes of amplicons for the deletions: AN3860 (WT - 1520 bp, mutant - 620 bp), AN10419 (WT - 1905 bp, mutant - 539 bp) and AN7230 (WT - 3653 bp, mutant - 539 bp). Southern Blotting (below) performed from the digested gDNA of the parental strain *A. nidulans* Δ*ku* and the derived mutants. Predicted sizes of the fragments targeted by specific probes: AN3860 (WT - 1917 bp, mutant - 1017 bp), AN10419 (WT - 4060 bp, mutant - 2725 bp) and AN7230 (WT - 5281 bp, mutant - 2167 bp). MW - molecular weight standards; ΔΔ - refers to mutants carrying double deletions; ΔΔΔ refers to mutants carrying triple deletions.

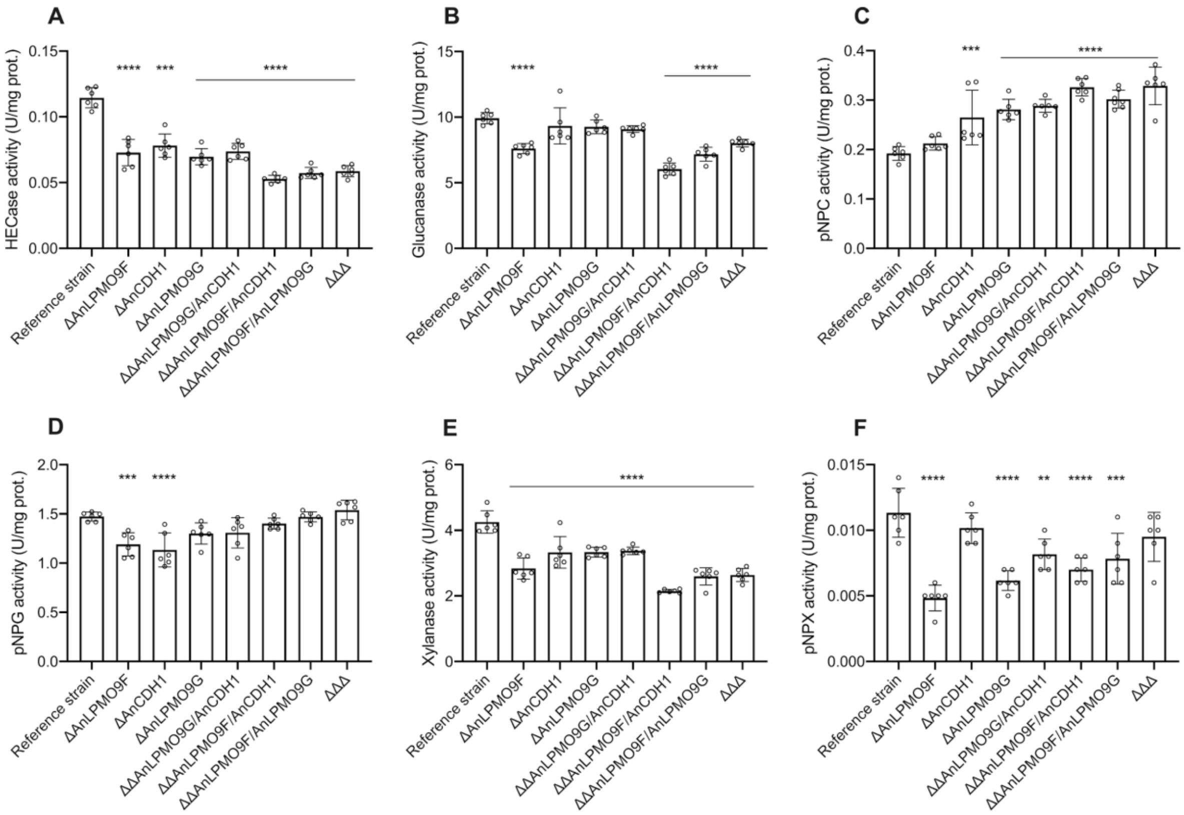


**FIG S3** Activity profile of *A. nidulans* mutants carrying single, double and triple deletions for *An*LPMO9F (AN3860), *An*LPMO9G (AN10419) and *An*CDH1 (AN7230). Activities were assayed using the secretomes induced on Avicel and the following substrates: (A) HEC, (B) β-glucan, (C) pNPC, (D) pNPG, (E) xylan and (F) pNPX. Error bars indicate standard deviations from six replicates. Statistics were taken from Dunnett's multiple comparisons used as follow up test to ANOVA. *$P < 0.05$; **$P < 0.01$; ***P<0.001; ****$P < 0.0001$.

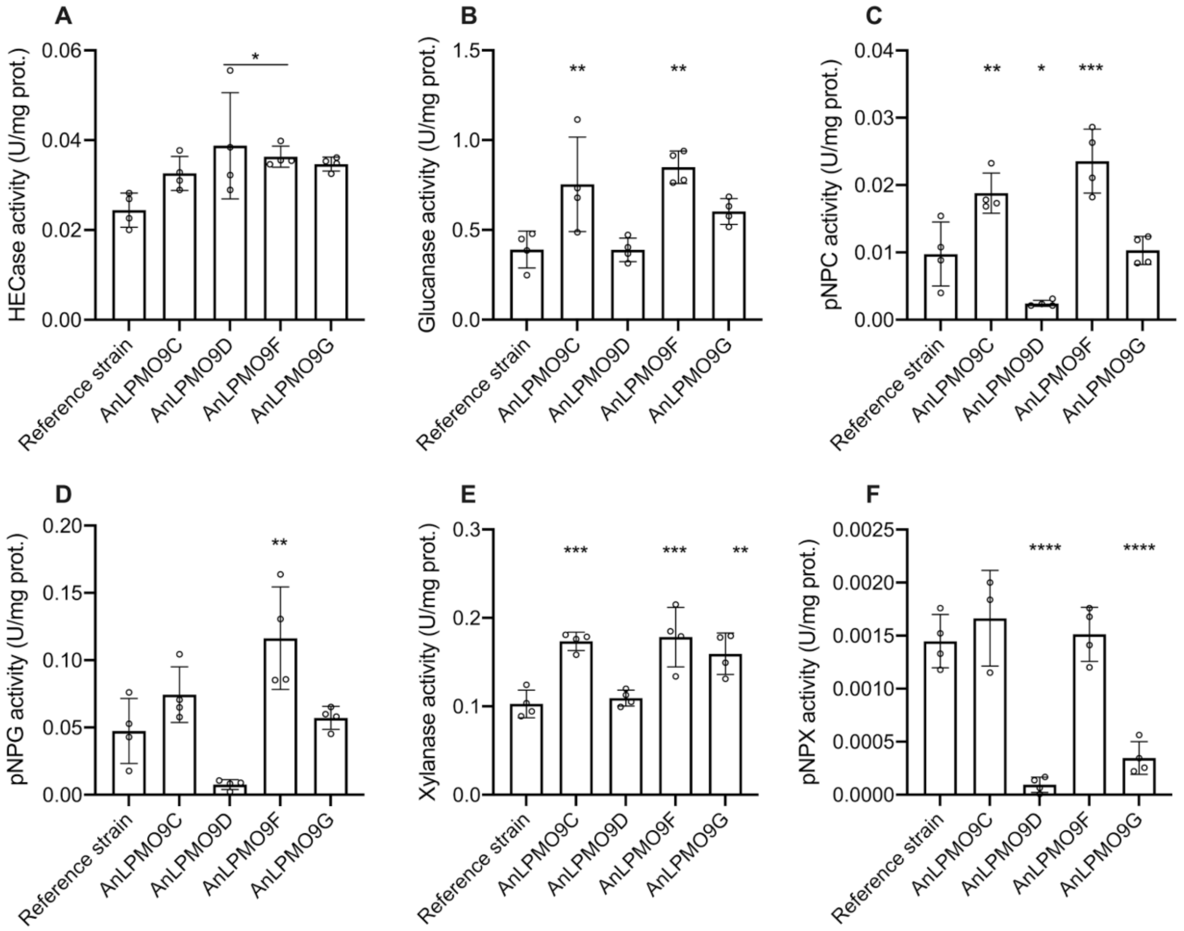


**FIG S4** Activity profile of the *An*LPMO9-enriched secretomes. The secretomes were obtained by performing cultivation of the *A. nidulans* strains overexpressing *An*LPMO9C (AN6428), *An*LPMO9D (AN3046), *An*LPMO9F (AN3860) and *An*LPMO9G (AN10419) in liquid minimal medium with Avicel followed by subsequent induction by maltose, conditions adapted for co-expression of cellulolytic enzymes and high levels of the targets *An*LPMO9. Activities were assayed using the following substrates: (A) HEC, (B) β-glucan, (C) pNPC, (D) pNPG, (E) xylan and (F) pNPX. Error bars indicate standard deviations from four replicates. Statistics were taken from Dunnett's multiple comparisons used as follow up test to ANOVA. *$P < 0.05$; **$P < 0.01$; ***$P<0.001$; ****$P < 0.0001$.

## List of supplementing tables

**Table S1 -** Mass spectrometry dataset - raw data of proteins identified by Orbitrap MS/MS in the extracellular proteomes from *A. nidulans* A773 cultivated in liquid MM with SCB, SCS, Avicel and glucose; 3 cultivation periods; 3 biological replicates.

**Table S2 -** Mass spectrometry dataset - raw data of proteins identified by Q-TOF MS/MS in the extracellular proteomes from *A. nidulans* Δ*ku* and derived mutants carrying single deletions for *An*LPMO9F (AN3860), *An*LPMO9G (AN10419) and *An*CDH1 (AN7230) cultivated in liquid MM with Avicel; one cultivation period (24 h); 3 biological replicates.

**Table S3 -** Fungal strains used in this work.

**Table S4 -** Primers and repairing oligosaccharides used in this work.
